# 352. Post-Discharge Microbiology Review by Infectious Diseases Pharmacists is Impactful

**DOI:** 10.1093/ofid/ofad500.423

**Published:** 2023-11-27

**Authors:** Amy Van Abel, Margaret Pertzborn, Trudi Lane, Diana Schreier, Hiliary R Teaford, Abinash Virk, Courtney Willis, Christina G Rivera (O'Connor)

**Affiliations:** Mayo Clinic, Lino Lakes, Minnesota; Mayo Clinic Health System, Eau Claire, Wisconsin; Mayo Clinic, Lino Lakes, Minnesota; Mayo Clinic, Lino Lakes, Minnesota; Mayo Clinic, Lino Lakes, Minnesota; Mayo Clinic, Lino Lakes, Minnesota; Mayo Clinic, Lino Lakes, Minnesota; Mayo Clinic, Lino Lakes, Minnesota

## Abstract

**Background:**

Post-discharge culture and susceptibility review has been described in emergency department and urgent care settings, but there is a lack of published literature describing culture review after acute care hospitalizations. Awaiting microbiology results can impede timely patient discharge. However, discharging patients while microbiology results are pending may result in suboptimal therapy or lost opportunity for additional testing/follow up. We implemented an antimicrobial stewardship effort to identify microbiology tests that result post-discharge utilizing a custom report in the electronic medical record (EMR).

***Methods.*** This retrospective descriptive study included adult patients ( >18 years of age) at 4 sites within Mayo Clinic from 1/1/2019 to 2/28/2023. Eligible patients had a hospitalization with an ID consult and an abnormal culture that was reviewed by an antimicrobial stewardship pharmacist after discharge. Urine cultures were excluded. The details of pharmacist-initiated antimicrobial stewardship interventions based on the culture review were identified by an EMR based research report.

**Results:**

A total of 6803 patient encounters with at least 1 microbiology result reviewed post-discharge were identified. Patients were 40% female with a median age of 62 years. Patient race was 89.8% white, 4% black/African American, 1.5% Asian, and < 1% other. Of these patients, 1983 (29%) had at least one pharmacist intervention. A total of 169 (2.5%) of these patients had multiple interventions. Interventions included modification of antimicrobial therapy, addition of susceptibility testing, or additional follow up (labs, imaging, or patient visits). Median time from culture update to pharmacist review was 27.2 hours. Median length of hospital stay was 5 days and discharge disposition was home with self care (65%), home with home health or hospice (22.7%), or discharge to another acute care or skilled nursing facility (12.3%).

**Conclusion:**

Outpatient review of post-discharge culture updates utilizing EMR reporting led to substantial interventions impacting antimicrobial treatments.

**Disclosures:**

**Christina G. Rivera (O'Connor), Pharm.D**, Gilead Sciences: Advisor/Consultant|Gilead Sciences: Board Member|Gilead Sciences: Grant/Research Support|Gilead Sciences: Honoraria

